# Crystal Structures of *Trypanosoma brucei* Oligopeptidase B Broaden the Paradigm of Catalytic Regulation in Prolyl Oligopeptidase Family Enzymes

**DOI:** 10.1371/journal.pone.0079349

**Published:** 2013-11-12

**Authors:** Peter Canning, Dean Rea, Rory E. Morty, Vilmos Fülöp

**Affiliations:** 1 School of Life Sciences, University of Warwick, Coventry, United Kingdom; 2 Department of Lung Development and Remodelling, Max Planck Institute for Heart and Lung Research, Bad Nauheim, Germany; NCI-Frederick, United States of America

## Abstract

Oligopeptidase B cleaves after basic amino acids in peptides up to 30 residues. As a virulence factor in bacteria and trypanosomatid pathogens that is absent in higher eukaryotes, this is a promising drug target. Here we present ligand-free open state and inhibitor-bound closed state crystal structures of oligopeptidase B from *Trypanosoma brucei*, the causative agent of African sleeping sickness. These (and related) structures show the importance of structural dynamics, governed by a fine enthalpic and entropic balance, in substrate size selectivity and catalysis. Peptides over 30 residues cannot fit the enzyme cavity, preventing the complete domain closure required for a key propeller Asp/Glu to fix the catalytic His and Arg in the catalytically competent conformation. This size exclusion mechanism protects larger peptides and proteins from degradation. Similar bacterial prolyl endopeptidase and archael acylaminoacyl peptidase structures demonstrate this mechanism is conserved among oligopeptidase family enzymes across all three domains of life.

## Introduction

African sleeping sickness (or human African trypanosomiasis) is a neglected disease affecting 60 million people in sub-Saharan Africa [1]. In some regions, prevalence has reached 50%, making it a greater mortality risk than HIV/AIDS [2]. Tragically, sufferers inhabit some of the poorest countries, and the low profitability of potential treatments or cures has resulted in the pharmaceutical industry choosing not to invest in much-needed research in this area. The only currently available drugs are highly toxic [2], [3], [4] but the disease is fatal if left untreated. This disease, caused by the protozoan parasite *Trypanosoma brucei* and spread by the bite of the tsetse fly [5], has proliferated across central and western Africa as the *Trypanosoma brucei gambiense* subspecies, and across eastern and southern Africa as the *Trypanosoma brucei rhodesiense* form. *T. brucei* is closely related to *Trypanosoma cruzi*, the causative agent of Chagas disease (human American trypanosomiasis), another neglected disease similarly transmitted by large blood-sucking parasites, the triatomines or kissing bugs [6]. Chagas disease affects around 11 million people in poorer parts of central and southern America, and is similarly fatal if left untreated.

It is therefore important to develop new, affordable treatments for these diseases. Oligopeptidase B (OPB, EC 3.4.21.83), an enzyme belonging to the prolyl oligopeptidase (PREP) family, has recently emerged as a virulence factor in *T. brucei* and other trypanosomes, and is a potential therapeutic target [7]. OPB hydrolyses peptide substrates of up to 30 amino acid residues in length, after arginine or lysine [8] with a preference for arginine [9] and most efficiently after a pair of basic residues [10]. The exact physiological roles and substrates for OPB remain unknown, but in *T. cruzi*, OPB is needed to generate mammalian host cell calcium-mediated membrane lysosome recruitment that the parasite then exploits to achieve cell invasion [11]. The *T. brucei* enzyme does not appear to have an identical role in cell invasion as *T. brucei* has no intracellular stage [12]. Rather, *T. brucei* OPB (TbOPB) appears to be released from disrupted dead or dying parasites into the bloodstream, where it is unregulated and remains active, disrupting host hormone signaling pathways that contribute to disease progression [13], [14]. Specifically, mutant parasites lacking OPB exhibited significantly higher levels of cysteine peptidase and prolyl oligopeptidase activity than wild type parasites [12]. Interestingly, TbPREP protein levels were not increased despite the increased PREP activity, suggesting that either a PREP-like enzyme compensates for a loss in OPB activity, or perhaps OPB is involved in generating an inhibitor of PREP that is decreased or absent in the OPB deficient parasites. OPB is similarly released into the bloodstream by *T. evansi* [13] and *T. congolense* [6]. A similar gene deletion study suggested OPB is not an essential virulence factor in *Leismania major* [14], a species belonging to a different class of protozoan parasites that cause leishmaniasis. This work also highlighted the possibility that a second OPB-like enzyme (OPB-2) may compensate in the OPB null mutant. OPB-2 from *Leishmania amazonensis* has been characterized and has all of the catalytic and substrate-binding residues, along with an unusual ∼200 residue C-terminal extension [15]. Despite uncertainties over the exact role of OPB in trypanosome physiology and pathology, the enzyme remains a strong candidate for therapeutic intervention, as shown by OPB inhibitors that kill cultured *T. brucei* parasites and cure infected mice [16]. Particularly effective inhibitors, at least *in vitro*, are tripeptide aldehydes with arginine residues at the P1 position, such as leupeptin and antipain [7], [17].

Structure-based drug design has advantages over traditional drug discovery and development procedures, and is becoming increasingly important in the development of novel pharmaceuticals [18]. This approach requires high-resolution experimentally determined structures from crystallography and NMR. The crystal structure of OPB from *L. major* (LmOPB) has recently been determined [19], but no structures of a trypanosomal OPB enzyme have been reported. LmOPB has the typical 2-domain α/β-hydrolase and β-propeller structure first observed in the crystal structure of the related PREP enzyme [20] and present in all members of the PREP family structurally characterized to date [21]. LmOPB was crystallised in the presence of the inhibitor and transition state analogue antipain, revealing the molecular mechanisms of substrate binding and specificity [19]. This information, whilst valuable, represents a single snapshot of the catalytic process; a closed state structure containing the inhibitor completely buried in the internal cavity of the enzyme. This snapshot doesn’t reveal the structure of the enzyme in the resting state, or how substrates and products enter and leave the active site. Structural dynamics are clearly important in regulating catalysis in this family of enzymes. Crystal structures of both bacterial PREP [22], [23] and archael acylaminoacyl peptidase (AAP) [24] in a ligand-free open state, in which the 2 domains are separated in a hinge-like manner, demonstrate the inherent flexibility of these molecules. These structures represent potential resting states, suggest domain separation as the mechanism of ligand entry and indicate an induced fit mechanism of substrate binding. However all mammalian enzyme crystal structures determined to date are closed state structures, even in the absence of a ligand [20], supporting a conformational selection mechanism of substrate binding. It has been suggested that mammalian PREP remains in the domains-together closed state throughout the catalytic cycle, with more subtle surface loop fluctuations sufficient for substrate entry [25], [26], although the requirement for inter-domain flexibility was previously shown using engineered disulfide bridges [27], [28]. Recent NMR relaxation experiments on mammalian PREP strongly favor a resting state consisting of an equilibrium of open and closed states [29]. Additional crystal structures of PREP family enzymes from eukaryotes, and further biophysical studies, are clearly needed.

Other PREP family enzymes have also been linked to roles in pathophysiological processes, and inhibitors are gradually making their way through clinical trials. PREP itself appears to be involved in processing signaling hormones and neuropeptides [30], has been linked to memory and learning [31], [32], and is a potential target for the treatment of cognitive disorders and neurodegenerative diseases [33], [34]. Dipeptidyl peptidase 4 (DPP4) is involved in the regulation of incretins GLP-1 and GIP that control blood sugar, and DPP4 inhibitors Sitagliptin, Vildagliptin and Saxagliptin are approved as drugs to treat type 2 diabetes [26]–[28]. This bodes well for OPB inhibitors as potential anti-parasitic drugs. To this end, we have determined crystal structures of *T. brucei* OPB in the ligand-free open state and antipain-bound closed state. These results provide important information for OPB inhibitor development, and broaden our understanding of the role of structural dynamics in the mechanism of catalytic regulation in PREP family enzymes.

## Results

### Overall Structure and Oligomerization

**Figure 1:**
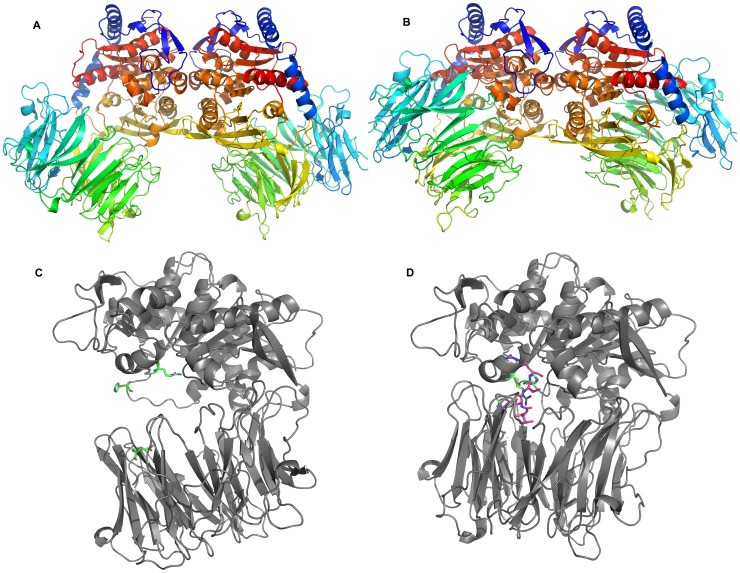
The TbOPB structure. A: Cartoon of the TbOPB open state dimer. The large distance between the α/β-hydrolase and propeller domains is apparent. B: Cartoon of the TbOPB-Antipain closed state dimer. The α/β-hydrolase and β-propeller domains are in much closer contact, effectively packed against each other. The α/β-hydrolase domains are in the same positions in (A) and (B) to facilitate easy visualization of the structural changes. The protein chains are coloured blue to red from the N to C termini. C, D: Comparison of the open (C) and closed (D) states of the enzyme. A single protein chain of each dimer is shown for clarity. Functionally important residues and loops that undergo localised structural changes between the open and closed states are coloured green, and the bound Antipain is coloured magenta.

Recombinant TbOPB was prepared and crystallized as previously described [35]. Crystal structures were determined in the ligand-free open state and inhibitor-bound closed state at resolution 2.4 and 2.85 Å, respectively (Table 1). Gel filtration chromatography previously indicated that TbOPB and the related TcOPB are dimeric in solution [36], [37], and this was confirmed recently for *T. cruzi* [38]. Protein-protein interfaces present in the crystal lattice may represent a true oligomer interface or merely a crystal packing contact involved in stabilizing the crystal lattice. The PISA server [39] analyses protein-protein interfaces present in the crystal lattice and predicts the likelihood of representing a genuine biologically relevant interface based on the amount of buried surface area and the theoretical free energy. In this study, PISA identified two potential dimer interfaces present in the TbOPB open state structure, each involving ∼1000 Å^2^ of buried surface area (Fig. S1A). One interface involved contacts between the ãβ-hydrolase domain of one molecule with the β-propeller domain of the neighboring molecule, and included a disulfide bridge between Cys169 of each molecule (Fig. S1B, C). PISA predicted this to be a true dimer interface with 100% confidence. The other potential dimer interface involved contacts between only the ãβ-hydrolase domains of neighboring molecules (Figs. S1A and 1A) and is predicted to be a crystal packing interface only. However PISA identified this same less favored interface in the TbOPB closed state crystal lattice (Fig. 1B), confirming this as the true dimer interface, whilst the other more favored interface is not present in the closed state structure. Interestingly, when Cys169 was mutated *in silico* to an Ala residue, or Thr residue as is present in TcOPB, PISA still favored this incorrect interface as the more probable, albeit with lower confidence. Without the closed state structure to compare with, it would have been easy to wrongfully assume that the lattice contact was the biological interface. With hindsight the lattice contact involving both domains and a disulfide bridge would impede the inter-domain flexibility needed to allow opening and closing of the enzyme during catalysis (discussed below), and therefore could not be the biological dimer interface. Furthermore, whilst dimerization was not mentioned or discussed in the recent LmOPB crystal structure [19], PISA analysis identified the same protein-protein interface in that crystal lattice of this structure, supporting dimerization via an equivalent physiological dimer in this related enzyme. In contrast to PISA, the EPPIC server [40] correctly identified the correct physiological dimer, whilst the crystallographic dimer was scored lower. EPPIC classifies protein-protein interfaces based on an evolutionary analysis, and may be more capable of distinguishing physiologically relevant interfaces from lattice contacts. This analysis proved to be a reminder of the current limitations of structure prediction programs, the preference for experimentally determined structures, and the importance of considering the biological and functional context of experimentally determined structures.

**Table 1:** Data collection, phasing and refinement statistics.

	*Open (Se-Met)*		*Closed*
**Data collection**			
Space group	P3_2_21		P2_1_
Cell dimensions			
*a*, *b*, *c* (Å)	124.14, 124.14, 249.14		71.8, 148.8, 268.0
α, β, γ (°)	90, 90, 120		90, 91.0, 90
	*Remote*	*Peak*	
Wavelength	0.9751	0.9789	0.9778
Resolution (Å)	66–2.4 (2.53–2.4)	66–2.4 (2.58–2.45)	59–2.85 (3.00–2.85)
*R*_sym_ a	0.167 (1.609)	0.186 (1.587)	0.219 (1.123)
*I*/ 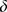 (*I*)	18.6 (2.0)	16.6 (2.2)	8.3 (2.2)
Completeness (%)	99.3 (95.6)	99.6 (97.5)	96.7 (97.7)
Redundancy	34.6 (22.2)	35.1 (28.2)	6.5 (6.6)
**Refinement**			
No. reflections	86984		127053
*R*_cryst_ b	0.183 (0.333)		0.213 (0.293)
Reflections used	83463 (5723)		121890 (8893)
*R*_free_ c	0.233 (0.383)		0.286 (0.396)
Reflections used	3521 (237)		5162 (379)
R_cryst_ (all data)^b^	0.185		0.216
No. atoms			
Protein	11302		3384
Ligand/ion	0		258
Water	778		344
*B*-factors			
Protein	52.1		47.2
Ligand			54.6
Water	49.9		33.2
R.m.s deviations			
Bond lengths (Å)	0.015		0.011
Bond angles (°)	1.8		1.7
**Molprobity scores**			
Clash score	7.6		15.4
Clash score percentile	98		96
Ramachandran space (%)			
Favored	95.8		88.2
Allowed	3.4		9.5
Outliers	0.8		2.3

Numbers in parentheses refer to values in the highest resolution shell.

a *R*_sym_ = Σ_j_Σ_h_|*I*_h,j_−<*I*_h_>|/Σ_j_Σ_h_<*I*_h_> where *I*_h,j_ is the jth observation of reflection h, and <*I*_h_> is the mean intensity of that reflection.

b *R*_cryst_ = Σ||*F*_obs_|−|*F*_calc_||/Σ|*F*_obs_| where *F*_obs_ and *F*_calc_ are the observed and calculated structure factor amplitudes, respectively.

c *R*_free_ is equivalent to *R*_cryst_ for a 4% subset of reflections not used in the refinement.

Dimerization is mediated through the burial of ∼1000 Å^2^ of hydrophobic surface area, and additionally through 2 salt bridges between both Asp621 and Arg633 residues of neighboring molecules. These salt bridges are conserved in the LmOPB structure. The dimer interface is necessarily distant from the interdomain interface and active sites, *and would therefore not be expected to influence the catalytic activity.* All further structure-function analyses are carried out on a single protein chain.

The overall structure of TbOPB comprises the typical 2 domain α/β-hydrolase and β-propeller architecture (Fig. 1C, D) that is present in all members of the PREP family structurally characterized to date [21]. A short N-terminal segment (residues 1–87) and long C-terminal segment (residues 436–714) together form the α/β-hydrolase domain, whilst the region in between (residues 88–435) forms the β-propeller domain. TbOPB shares 60% sequence identity with LmOPB. The TbOPB-antipain closed state structure aligned with the equivalent LmOPB structure [19] with a root mean square deviation (RMSD) of 0.59 Å for 588 CA atoms. The secondary structure is therefore very similar to that of LmOPB described previously [19].

### Active Site and Substrate Binding

**Figure 2:**
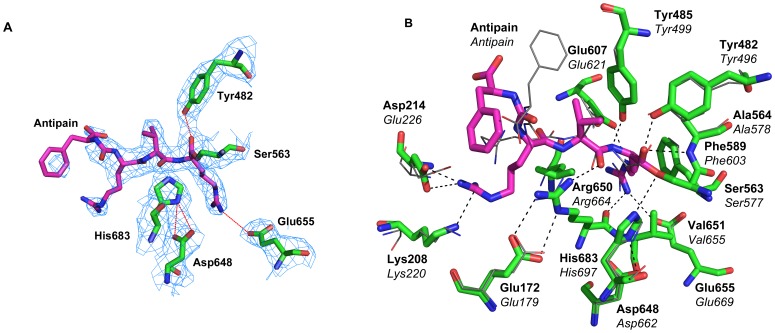
The catalytic site of TbOPB. A: Electron density of the active site of the TbOPB-Antipain closed state complex, contoured at the 0.8 σ level, where σ represents the RMS electron density for the unit cell. Contours more than 1.4 Å from any of the displayed atoms have been removed for clarity. TbOPB carbon atoms are coloured green, and Antipain carbon atoms are coloured magenta. B: Detailed view of the active site of the TbOPB-Antipain closed state complex. The catalytic triad Asp648, His683, and Ser563 form a charge relay. Ser563 forms a covalent hemiacetal transition state analogue complex with Antipain. The oxyanion is stabilized by Ala564 and Tyr482. The P1 Arg is bound to Glu607, Glu655, Arg650, and has a π-stacking interaction with Phe589. The P2 carbonyl is hydrogen bonded to Arg650. The LmOPB-antipain structure (PDB code 2XE4) determined previously [19] is superimposed and is shown in line representation. The P3 Arg interacts with Ser523 and Leu617, whilst in TbOPB interactions are with Asp214 and Lys208 of the propeller domain. The P4 Phe does not make any specific interactions with enzyme residues in either TbOPB or LmOPB. TbOPB and LmOPB residues are labeled in bold and italic type, respectively.

**Figure 3:**
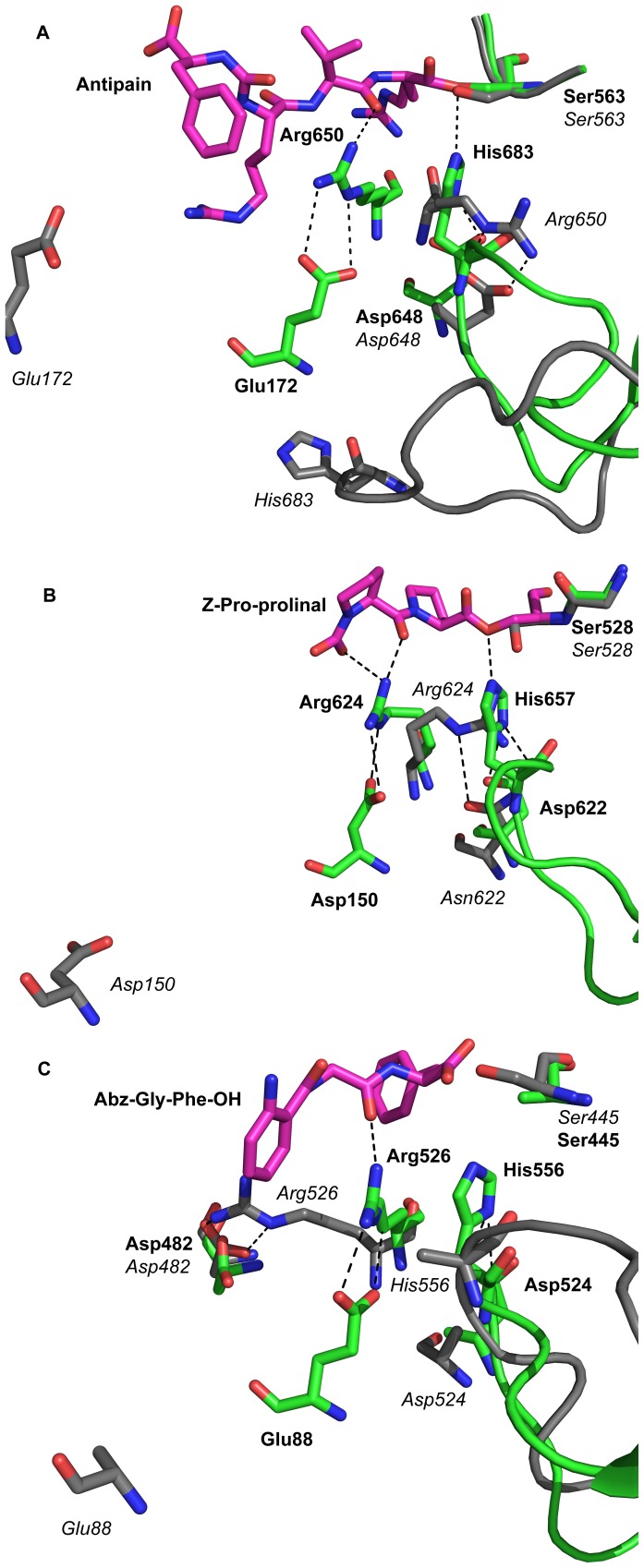
Comparison of the catalytic site in open and closed structures of prolyl oligopeptidase family enzymes. A: Detailed view of the active site of TbOPB in the open and closed state. Open and closed states are superposed to clearly show the localised structural changes that occur upon substrate binding/domain closure. Open state residues are coloured grey, closed state residues are coloured green, and the bound Antipain is coloured magenta. In the open state, the propeller domain Glu172 is a long distance from the active site residues. Arg650 is in a catalytically incompetent conformation in which it binds to the catalytic triad Asp648, displacing the catalytic triad His683 and the His loop from the active site, rupturing the catalytic triad and inactivating the enzyme. Upon domain closure, Glu172 is brought into the active site, where it pulls Arg650 into the catalytically competent position, away from the catalytic triad Asp648, which allows His683 and the His loop to swing into the active site, where His683 can complete the catalytic triad and activate the enzyme. B: ApPREP open (PDB code 3IUN) and closed (PDB code 3IVM) structures. C: ApAAP open (PDB code 3O4J) and closed (PDB code 2HU8) structures. Open and closed states are coloured, superposed, and shown in the same orientation as (A) to facilitate comparison between the different PREP family enzymes. Very similar global and local conformational changes are apparent in these structures, supporting a conserved mechanism of catalytic regulation in different PREP family enzymes. Residues in the open and closed structures are labeled in italic and bold type, respectively.

TbOPB was cocrystallised with the inhibitor antipain to investigate the structural features responsible for substrate recognition, specificity and catalysis. Electron density was visible for all active site residues and the covalently bound antipain inhibitor (Fig. 2A). Whilst broadly similar to the equivalent LmOPB-antipain complex structure (shown in line representation) [19], there are differences in the P3 and P4 positions of the bound ligand, and some additional enzyme-inhibitor interactions are observed (Fig. 2B). The catalytic triad Asp648, His683, and Ser563, form the charge relay and Ser563 has reacted with the P1 aldehyde of antipain to form a covalent hemiacetal transition state analogue complex. The oxyanion is stabilized by hydrogen bonding to the NH group of Ala564, the residue that follows the catalytic Ser in the sequence, as is characteristic of α/β-hydrolase enzymes. The oxyanion is further stabilized by hydrogen bonding to the OH group of Tyr482, as observed for LmOPB and the related PREP [20] and DPP4 [41], but not AAP [42] that instead has another hydrogen bond to a main chain NH (Fig. 2B). OPB specifically hydrolyses peptides at basic residues, with hydrolysis occurring fastest after a dibasic pair, and Arg is the preferred residue at the P1 and P2 positions [43]. The primary specificity for a P1 Arg is provided by specific interactions with Glu607 and Glu655 (Fig. 2B). The main chain carbonyl of Arg650 also contacts the guanidinium group of the P1 Arg, and additional specificity is provided by a π-stacking interaction with the side chain of Phe589 (Figs. 2B and 3A). Antipain has a Val at the P2 position. Whilst no obvious acidic residues that could interact with a preferred Arg at the P2 position were evident in the structure, Tyr485 may be well positioned to interact with a P2 Arg via a similar π-stacking interaction to that seen between Phe589 and the P1 Arg (Fig. 2B). The P2 carbonyl is hydrogen bonded to the important Arg650, and this interaction is also conserved in PREP (Fig. 3B) and AAP (Fig. 3C). The position of the P3 Arg of the bound ligand differred in the OPB structures. In LmOPB the P3 Arg interacts with Ser523 and Leu617, whilst in TbOPB an alternative conformer was adopted, resulting in interactions with Asp214 and Lys208 of the propeller domain (Fig. 2B). This may represent a degree of flexibility at this position of the substrate. Finally, the P4 Phe also adopted different positions in the two structures, but faced the enzyme cavity in both, and made no specific interactions with enzyme residues in either conformation. Nevertheless, the electron density at the P4 position is weak, indicating flexibility and multiple conformations.

### Open and Closed State Structures

The overall open and closed TbOPB structures (Fig. 1C, D) illuminated the hinge-type motions that facilitate domain separation during catalysis. The peptidase domain of the open and closed structures superimposed with an RMSD of 0.37 Å for 291 CA atoms, whilst the propeller domains aligned with an RMSD of 0.5 Å for 309 CA atoms. The domains therefore move predominantly as rigid bodies during opening and closing of the molecule. Although apparently subtle, the more localized structural differences between individual amino acid residues in the open and closed state are significant, and are the key to understanding the mechanism of catalytic regulation in OPB and the wider PREP enzyme family (discussed below). The most obvious difference is the distance between the two domains (Fig. 1C, D). Domain separation pulls the propeller residue Glu172 out of and away from the active site. Whilst not directly involved in catalysis itself, this residue is responsible for fixing the crucial Arg650 in the appropriate catalytically active conformation in the closed state. In the open state, and in the absence of Glu172, Arg650 adopts a quite different position in the active site, binding instead to the catalytic triad Asp648, which in turn displaces the catalytic triad His683 to the surface of the molecule (Fig. 3A). This swing of the catalytic His683 loop involves a movement of 11 Å for His683 itself (as measured for the CA atoms of the open and closed state structures. This disrupts the catalytic triad, rendering the enzyme inactive in the open state.

## Discussion

### Structural Comparison with PREP and AAP

Open and closed state crystal structures have now been determined for OPB and the related PREP family enzymes PREP and AAP, but the electron density was unfortunately incomplete for the more mobile and functionally important parts of the open state structures of these enzymes, and this has prevented a complete visualization and appreciation of the conformational changes that occur through domain opening and closing during catalysis. Fortuitous crystal contacts have presumably stabilized these more mobile regions in the TbOPB open state structure, resulting in complete electron density for these previously disordered regions. The structural differences between the key active site residues in the open and closed states of OPB, PREP and AAP are clearly conserved (Fig. 3). The DynDom server [44] was used to compare TbOPB and *Aeropyrum pernix* AAP (ApAAP) open and closed structures. Both enzymes clearly open to a similar extent; TbOPB exhibited a domain rotation of 27.8° between the open and closed structures, whilst the corresponding values for the two dimers in the ApAAP structure (PDB code 3o4j) were 27.6 and 28.2°, respectively. The TbOPB open and closed structures are therefore highly similar to the equivalent ApAAP structures, with comparable domain movements and domain rotations. In OPB, PREP and AAP, domain opening removes the key propeller Glu/Asp residue from the active site (Fig. 3). When this occurs, the key active site Arg residue, crucial for substrate binding, is no longer held in place by the propeller Asp/Glu, and is rendered more flexible in the open state. This Arg residue rotates within the active site to adopt an inactive conformation that displaces the catalytic triad His and the loop carrying this residue from the active site, thus rupturing the catalytic triad and inactivating the enzyme, as discussed above. In the case of OPB (Fig. 3A) and PREP (Fig. 3B) the Arg displaces the catalytic His directly to bind the catalytic Asp itself. The situation is a little more complicated for the AAP open state structures (Fig. 3C), of which there are nine subtly different structures in different subunits of the asymmetric unit of different crystal structures [24]. Some have only partial electron density for the catalytic Arg or His. Furthermore, three different conformations of His, and two different conformations of Arg, have been captured in different crystal packing interactions (one of which is shown in Fig. 3C). In some AAP open state molecules, Arg swings away from the catalytic Asp to form a salt bridge with Asp482 near the surface of the molecule. The different structures observed in the open state of AAP, whilst not identical to those observed for OPB and PREP, similarly signify an increased flexibility of the active site Arg and catalytic triad His in the open state that result from domain separation and consequent removal of the propeller Glu/Asp from the active site. The conserved nature of the conformational changes is most apparent when the key residues are superposed (Fig. S2); the structural alignment of the open (Fig. S2A) and closed (Fig. S2B) structures of OPB, PREP and AAP is striking.

The very similar open and closed state structures for OPB, PREP and AAP strongly support a divergent evolution within the PREP enzyme family, in which an ancestral enzyme (not necessarily an oligopeptidase) with an ãβ-hydrolase and a β-propeller, and a similar opening/closing mechanism, diverged to give the PREP, OPB and AAP lineages. However it should be noted that a convergent evolution mechanism for the three lineages, whilst less likely, can not be ruled out entirely given the available evidence.

A PISA analysis of the peptidase and propeller domain interface in the open and closed state structures of TbOPB, *Aeromonas punctata* PREP (ApPREP) and ApAAP was performed to identify any evolutionarily conserved features that could shed light on the structural changes and mechanism (Table 2). TbOPB (715 residues, 80,7 kDa), ApPREP (690 residues, 76.5 kDa), and ApAAP (659 residues, 73.9 kDa) bury 2160 Å^2^, 2325 Å^2^, and 2067 Å^2^ of surface area, respectively, in the interface between the peptidase and propeller domains in the closed state. Upon opening, 56%, 46% and 51% of the surface area buried in the closed state remains buried in the open state for TbOPB, ApPREP and ApAAP, respectively. Thus between 44% and 54% of the buried surface area is lost upon opening. Further, between 53% and 65% of the inter-domain hydrogen bonds are lost upon opening, and between 38% and 100% of the salt bridges. Despite being 6% larger than ApPREP in molecular weight, TbOPB has an inter-domain interface that buries 7% less surface area. The much larger number of inter-domain hydrogen bonds/salt bridges in ApAAP likely reflects the fact that *Aeropyrum pernix* is an extremophile that lives and grows between 70–100°C. Given the relatively smaller size of ApAAP, the expected number of hydrogen bonds in the closed state would be 25–27 rather than the observed 49, and 4–6 salt bridges rather than the observed 14. The greater thermal motion at these temperatures results in much greater conformational flexibility/entropy for the thermophilic enzyme. The increased number of inter-domain interactions may be needed to compete with this high entropy in order to ensure that the molecule samples the active closed state conformation often enough, and for long enough, to allow the mechanism of conformational selection (discussed below) to occur. The large number of interactions is necessary to persuade the molecule to sample the entropically less favorable closed state during structural fluctuations.

**Table 2:** PISA analysis of the peptidase and propeller domain interface in the open and closed states of TbOPB, ApPREP and ApAAP.

	TbOPB		ApPREP		ApAAP	
Number of Residues	715		690		659	
Molecular Weight (kDa)	80.7		76.5		73.9	
	**Closed Structure** **(4BP9)**	**Open Structure** **(4BP8)**	**Closed Structure** **(3IVM)**	**Open Structure** **(3IUN)**	**Closed Structure** **(2HU8)**	**Open Structure** **(3O4J)**
Buried Surface Area (Å^2^)	2160	1218 (56%)	2325	1061 (46%)	2067	1050 (51%)
Hydrogen Bonds	30	14 (47%)	26	9 (35%)	49	19 (39%)
Salt Bridges	8	5 (62%)	5	0 (0%)	14	2 (14%)

PDB codes for the analyses are shown. These are the same as those used in Fig. 3.

### A Common Mechanism of Catalytic Regulation in PREP Family Enzymes

Porcine PREP was the first member of the PREP enzyme family to be structurally characterized [20], and the human enzyme crystal structure has also been determined [45]. All mammalian PREP crystal structures determined to date are in the closed state, even in the absence of a ligand. These closed state structures don’t reveal an obvious passage for substrate access to the buried active site. Various potential substrate access routes were proposed and investigated, including through the pore at the base of the propeller through separating propeller blades [27], surface loop movements at the domain interface [46], and domain separation [28]. The crystal structure of bacterial *Sphingomonas capsulata* PREP in an open state was the first direct demonstration that domain separation may provide a route for substrate access [22]. It was proposed that ScPREP resides in a closed resting state, and the presence of substrate induces domain separation to allow substrate access to the active site. More recent open state crystal structures of bacterial *Aeromonas punctata* PREP were recently reported [23]. Upon soaking inhibitor Z-Pro-Prolinal (ZPP) into these crystals and collecting diffraction data, the resulting structure showed inhibitor binding and domain closure had occurred. An open resting state and an induced fit mechanism of substrate binding were proposed. Recent crystal structures of the archaeal *Aeropyrum pernix* AAP showed that different subunits can adopt open or closed states simultaneously in the crystal [24]. Indeed, in some crystals one subunit of the dimeric molecule is in the open state whilst the other subunit is in the closed state. It was concluded that AAP resides in an inherently flexible resting state that samples both the fully open and closed conformations during normal molecular fluctuations. The closed state is in a catalytically active conformation, even in the absence of ligand, as observed previously for mammalian PREP. This crystallographic evidence alone supports a conformational selection substrate binding mechanism. NMR studies on human PREP provide additional strong evidence for a conformational selection mechanism. ^15^N labeling of Trp residues demonstrated multiple conformations of ligand-free PREP in solution [47]. Site-directed mutagenesis coupled with ^15^N relaxation experiments in the presence and absence of the covalent inhibitor ZPP mapped the largest structural changes to Trp residues close to the inter-domain interface [29]. These findings strongly support an inherently flexible enzyme that rapidly samples a range of conformational states including the fully open and closed states that have previously been captured crystallographically for bacterial PREP [23], archael AAP [24], and in this work for OPB from a eukaryote. This implies a low energy barrier for the transition between the conformational states. This may at first seem counterintuitive, given the much greater buried surface area and increased number of hydrogen bonds/salt bridges in the closed state (Table 2). Indeed this is the main reason for disfavoring a mechanism involving domain separation rather than more subtle surface loop movements to allow substrate access [25], [48]. It appears that the higher enthalpy associated with the increased number of favorable contacts in the closed state is counterbalanced by the higher entropy associated with the more flexible open state. The much higher than expected number of inter-domain contacts in the hyperthermophilic ApAAP closed state structure supports this conclusion; these are needed to compensate for the increased conformational flexibility at the higher temperature, allowing the conformational sampling of the active closed state to occur for long enough to facilitate catalysis. It is conceivable that the inherent flexibility in these enzymes is used to manipulate the strength of specific interactions throughout the catalytic cycle, facilitating differential interactions with substrates, transition states and products, as well as substrate entry and product. After all, enzymes have evolved to catalyze multi-step reactions, not just to bind substrates or transition states, and this necessarily involves a subtle molecular plasticity that is difficult to comprehend from the static snapshots obtained from crystal structures and other biophysical studies. Experimental data supporting a low energy barrier for opening and closing are currently lacking. Sophisticated isothermal titration calorimetry (ITC) and differential scanning calorimetry (DSC) experiments coupled with site directed mutagenesis may shed light on the enthalpic and entropic balance associated with the equilibrium.

To conclude, evolution has selected for and finely tuned the enthalpic and entropic properties of these enzymes to engineer an innovative mechanism of substrate size selection that enables the fine control of cellular proteolysis needed to avoid proteolytic degradation of large peptides and proteins. Complete domain closure is required to bring the key propeller Asp/Glu residue into the active site to facilitate correct positioning of the key active site Arg and catalytic triad His. This domain closure can only occur with peptide substrates shorter than 30 residues due to the limited size of the enzyme cavity. Up to now, inhibitors of these enzymes have targeted the closed state and are predominantly peptidomimetics that may exhibit selectivity or bioavailability issues. Developing inhibitors targeted to the open state or hinge region, or that interfere with the structural dynamics of domain opening/closure, may provide an alternative therapeutic strategy to manipulate the catalytic activity and possible protein-protein interactions of OPB and other PREP family enzymes, and is sure to be an active area for future research.

## Materials and Methods

### Expression and Purification of Recombinant Trypanosoma brucei OPB

Recombinant *T. brucei* OPB was expressed and purified as previously described [35]. Selenomethionine-substituted protein was prepared for experimental phasing since no structures of sufficient similarity were available for structure determination by molecular replacement at the time. Labelled protein was expressed using *E. coli* strain B834 in 2 L conical flasks using established auto-induction methods [49], [50]. Selenomethionine-substituted protein was purified in the same way as the unlabeled enzyme [35], with the addition of 2 mM β-mercaptoethanol in all buffers to avoid oxidation of the selenium. Mass spectrometry confirmed the successful replacement of sulfur with selenium.

#### Crystallization and data collection

Crystallization was carried out using previously established crystallization conditions as a starting point [35]. Crystals were grown by the hanging-drop vapour diffusion method. Large, rice-shaped crystals grew in 9% (w/v) polyethylene glycol 6000, 1 M lithium chloride, 100 mM Bis-Tris propane, pH 7.5. Several crystals were removed from the crystallization drop using a nylon loop, cryoprotected in crystallization solution containing 15% glycerol, and flash-cooled in liquid nitrogen. Crystals of selenomethionine-substituted OPB were tested for X-ray diffraction on beamline ID-29 at the ESRF, Grenoble. A wavelength scan showed a strong anomalous signal at the selenium edge, further confirming successful selenium incorporation. Single wavelength anomalous dispersion (SAD) datasets were collected at the peak and high-energy remote wavelengths, in that order, to a resolution of 2.4 Å using a MAR CCD image plate (Table 1). Crystals containing the inhibitor antipain were prepared by mixing recombinant OPB at a concentration of 12 mg/mL, with 1 mM antipain. This mixture was subjected to an extensive screening regime using commercially available screens and a Cartesian Honeybee crystallization robot to set-up nL-scale sitting drops. After optimization using larger hanging drops, suitable crystals were grown in a solution containing 10% PEG 4000, 0.2 M calcium acetate, 0.1 M sodium acetate pH 5. Crystals were cryoprotected in crystallization solution containing 15% glycerol, and flash-cooled in liquid nitrogen. Diffraction data were collected to a resolution of 2.85 Å on beamline IO4 at Diamond synchrotron, UK, using an ADSC Q105 CCD detector (Table 1).

### Structure Determination, Model Building and Refinement

At this stage the LmOPB crystal structure [19] was not yet published, and using the closest available homologue structure (PREP) as a search model to solve the structure by molecular replacement proved unsuccessful. Selenomethionine-substituted OPB diffraction data were processed using MOSFLM [51] and SCALA [52]. The best phasing statistics, as judged by the resulting Rsym values, were achieved using only the data collected at the peak wavelength, and phasing proceeded by the single-wavelength anomalous dispersion (SAD) method using the peak wavelength data. The program SOLVE [53] successfully found 17 of the 18 selenium sites present in each molecule (the first Met is disordered in each subunit). RESOLVE [53] subsequently automatically fitted 82% of the total number of residues in the resulting electron density. Further refinement using the CCP4 suite of programs [54] using the high-energy remote data to be the best statistically, and was used to build the final model. BUCCANEER [55] was used for automated structure building, and the resulting model was improved by iterative rounds of refinement and model building using REFMAC5 [56] and COOT [57] or O [58]. Water molecules were added to the atomic model automatically using ARP-w-ARP [59] at the positions of large positive peaks in the difference electron density, only at places where the resulting water molecule fell into an appropriate hydrogen bonding environment. Refinement of the structure was carried out using noncrystallographic symmetry restraints. The OPB-antipain data were processed and scaled using XDS [60] and the final OPB model was used to determine the structure of the OPB-antipain complex by molecular replacement using PHASER [61]. This structure was similarly refined using REFMAC [56]. Refinement statistics are given in Table 1. Final models were visualized in, and figures produced using, PyMol [62]. The crystallographic asymmetric unit contains a dimer with residues 3–714 in the open structure. The density was interpretable for residues 5–714 for all three dimers in the crystallographic asymmetric unit in the closed structure.

#### Accession codes

The Protein Data Bank accession numbers for the ligand-free open state and inhibitor-bound closed state are 4BP8 and 4BP9, respectively.

## Supporting Information

Figure S1 **The nonphysiological crystallographic dimer in the TbOPB open structure.** A: Dimer of dimers looking down the crystallographic two-fold axis. The Cys169-Cys169 disulfide bridge between molecules of the crystallographic dimer is shown in stick representation. Each physiological dimer is comprised of one cyan (chain A) and one green (chain B) molecule. B: The crystallographic dimer showing only one subunit from each of the physiological dimers for clarity. C: Electron density of the disulfide bridge, contoured at the 1.0 σ level, where σ represents the RMS electron density for the unit cell. Contours more than 1.4 Å from any of the displayed atoms have been removed for clarity.
(TIF)

Figure S2 **Superimposition of TbopB, ApPREP and ApAAP open and closed structures.** A: Superimposition of TbOPB (PDB code 4BP8), ApPREP (PDB code 3IUN) and ApAAP (PDB code 304J) open structures. The orientations are the same as in Figure 3. Some of the residues are completely or partially disordered in the open structures. B: Superimposition of TbOPB (PDB code 4BP9), ApPREP (PDB code 3IVM) and ApAAP (PDB code 2HU8) closed structures. Parts A and B clearly show that the movements of the three key residues (propeller Asp/Glu, catalytic Arg and His) are conserved among the three different PREP family lineages. Carbons are green, cyan and orange for TbOPB, ApPREP and ApAAP, respectively, and magenta for the bound ligands. TbOPB, ApPREP and ApAAP residues are labeled in bold, italic and normal type, respectively.
(TIF)
