# Supplementary figures and images for: Crystal Structures of Trypanosoma brucei Oligopeptidase B Broaden the Paradigm of Catalytic Regulation in Prolyl Oligopeptidase Family Enzymes
Source: PLoS One. 2013 Nov 12;8(11):e79349. doi: 10.1371/journal.pone.0079349 (PMC3827171; doi:10.1371/journal.pone.0079349)

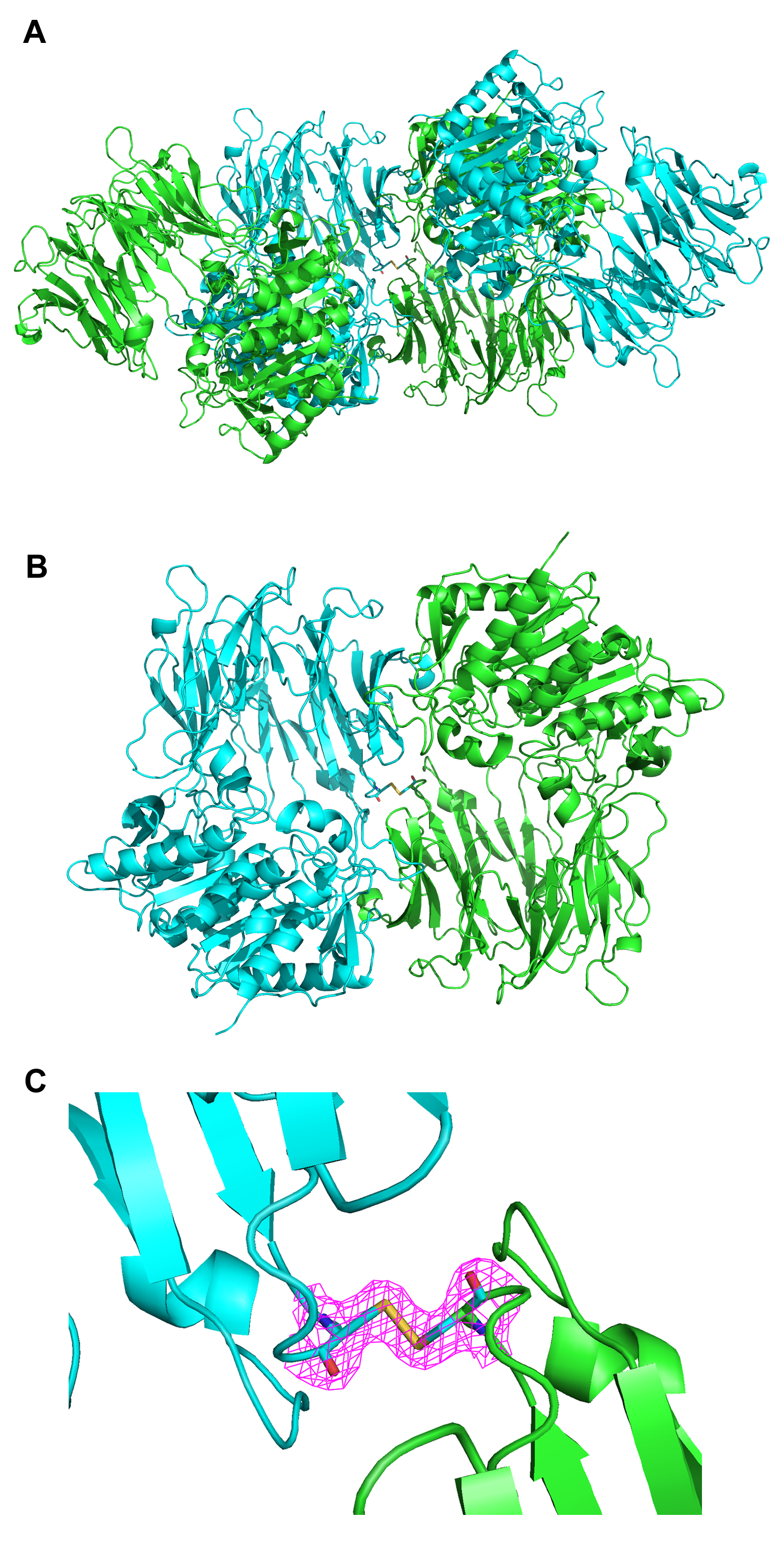

Supplement: Figure S1 — The nonphysiological crystallographic dimer in the TbOPB open structure. A: Dimer of dimers looking down the crystallographic two-fold axis. The Cys169-Cys169 disulfide bridge between molecules of the crystallographic dimer is shown in stick representation. Each physiological dimer is comprised of one cyan (chain A) and one green (chain B) molecule. B: The crystallographic dimer showing only one subunit from each of the physiological dimers for clarity. C: Electron density of the disulfide bridge, contoured at the 1.0 σ level, where σ represents the RMS electron density for the unit cell. Contours more than 1.4 Å from any of the displayed atoms have been removed for clarity. (TIF) [file pone.0079349.s001.tif]

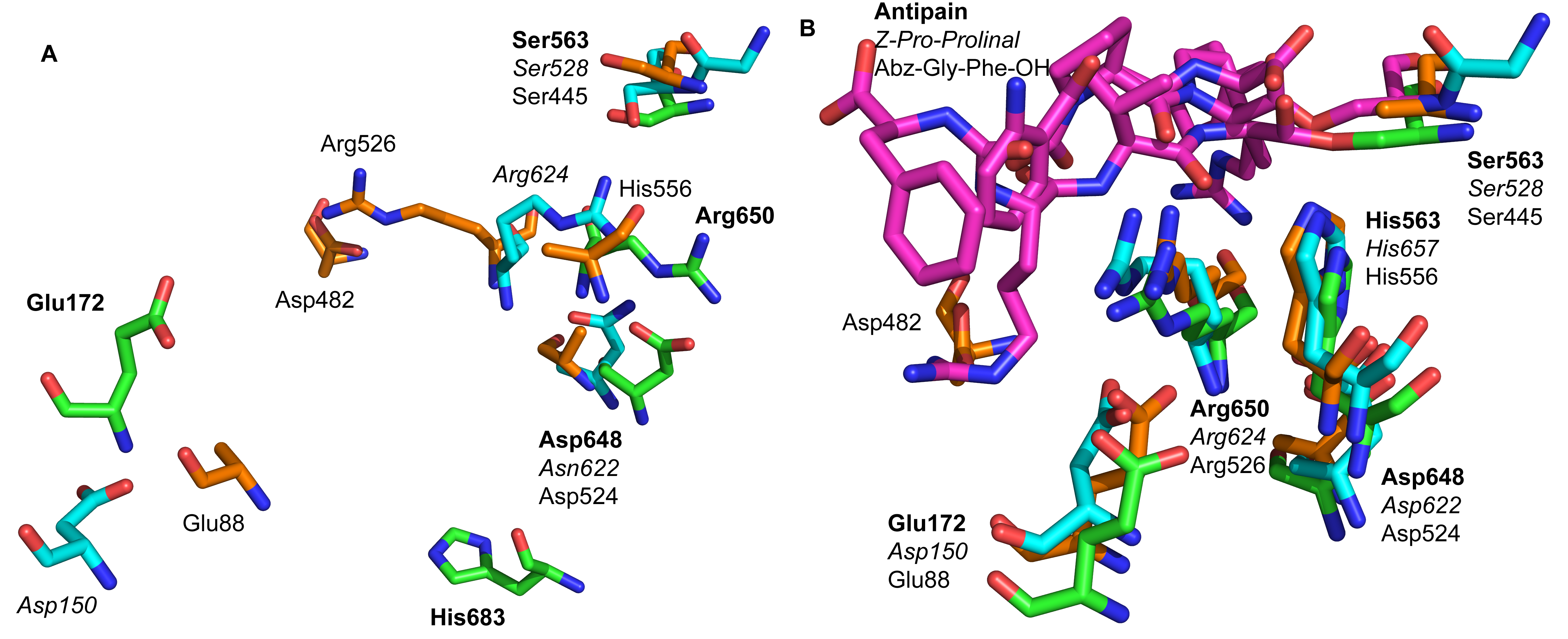

Supplement: Figure S2 — Superimposition of TbopB, ApPREP and ApAAP open and closed structures. A: Superimposition of TbOPB (PDB code 4BP8), ApPREP (PDB code 3IUN) and ApAAP (PDB code 304J) open structures. The orientations are the same as in Figure 3. Some of the residues are completely or partially disordered in the open structures. B: Superimposition of TbOPB (PDB code 4BP9), ApPREP (PDB code 3IVM) and ApAAP (PDB code 2HU8) closed structures. Parts A and B clearly show that the movements of the three key residues (propeller Asp/Glu, catalytic Arg and His) are conserved among the three different PREP family lineages. Carbons are green, cyan and orange for TbOPB, ApPREP and ApAAP, respectively, and magenta for the bound ligands. TbOPB, ApPREP and ApAAP residues are labeled in bold, italic and normal type, respectively. (TIF) [file pone.0079349.s002.tif]
